# On the Treatment of Aneurism

**Published:** 1849-11

**Authors:** 


### REVIEWS.

---

Art. II.—*On the Treatment of Aneurism.* By Benj. W. Dudley, M. D., etc., Professor of Surgery in Transylvania University. Translylvania Medical Journal. Vol. 1, No. 1. 1849.

Our readers have been made acquainted with the fact that another competitor for the favor of the medical public has made its appearance in the form of a bi-monthly, bearing the title of "Transylvania Medical Journal, edited by Prof. E. L. Dudley, under the supervision of the Transylvania Faculty of Medicine. This enterprise was announced in one of the Lexington papers sometime since and attracted our attention at the time. We have ever thought that Transylvania was in duty bound to sustain a medical periodical, which might serve as an exponent of the peculiar doctrines which, for so many years we have been informed are taught in that Mother of Western Medical Schools, and we have been at a loss whether to attribute the absence of such an exponent to poverty, parsimony, indolence or the lack of suitable materials on the part of those most interested in the prosperity of the Medical Department of Transylvania. But a variety of causes have conspired to depurate in a measure the old institution, and an infusion of young blood into the veins of her staunch old frame has been effected which, we think, will prove salutary—in the course of time.

The bi-monthly is one of the good effects of their infusion—a positive good which the old stagers would not undertake, or could not sustain, requiring, as it does for its successful execution, the energy, industry and genius of its projector and editor. A little more depuration and a little more infusion can do no hurt. We have so much confidence in the restorative influence of the remedy that, in this instance, we would hazard a little more purgation with the now enfeebled and tottering old patient, with the fullest assurance that if no good was done, no evil could result. When one has fallen to the foot of the hill he can tumble no lower.

However unfitted those, on whom time has left the deep impress of his heavy foot-fall, may be to receive, extend and enforce the important discoveries and improvements of modern times, they may yet be useful to us in imparting the light of other days—in detailing the conflicts of youth and portraying the victories of riper years. In this view all will be delighted to learn, as announced in the "Transylvania Medical Journal," that "a series of surgical essays will be commenced immediately, contributed to our pages by Prof. B. W. Dudley, which, when completed, will present a full record of his extensive experience, in that department of the profession."

This annunciation carries with it more weight from the fact that Prof. D. has long been regarded as the father of Western Surgery, and more especially because the profession at large have been taught to believe that his practice is distinguished alike by originality, simplicity, and extraordinary success. The desire, so long entertained, to have his views in a permanent form is about to be gratified; and as many of our readers may not meet with the journal in which they are to appear, we have thought that a brief synopsis of the essays, as they are published, might not be unacceptable. We have, therefore, concluded to furnish such a synopsis of the first of the series which has already appeared, and should it prove acceptable, and the duties of an active practice permit, we shall follow the Professor in the same manner until the whole is completed. The merits of the distinguished Professor as an original thinker, a well informed teacher, and successful practitioner we will seek to place on as high grounds as truth shall permit; and while we endeavor to separate the true from the false—the old from the new—and sophistry from reason, we shall strive to do no injustice to the venerable writer and to permit none to be perpetrated on others.

The title of the first paper of the series stands at the head of this article and will at once arrest the attention of the reader, as embracing a subject worthy the most serious consideration of the practical surgeon.

The learned professor falls into the common error of attributing to Mr. Hunter the honor of first suggesting the best and most successful mode of treating aneurism, which consists in the application of "a single ligature placed upon the artery at a remote point above the aneurism." This mode of treatment was proposed, and successfully practiced, two hundred years before it was adopted and taught by Hunter, as any one must be convinced by the perusal of the following extract from the writings of Ambroise Paré.

"Partant le conseille au jeune Chirurgien qu'il se garde d'ouvrir les aneurismes, si elles ne sont fort petites et en parties non dangereuse: coupant le cuir au dessus, le separant de l' artere, puis on possera une aiguille â seton, enfiléé d'un fort fil, par sous l' artere aux deux costés de la playe, et sera ladite artere liée, puis coupée, et la playe traitée, laissant tomber le filet de say-mesme: et se fasant nature engendre chair, qui sera cause de boucher l' artere."*

No one can doubt that this passage, the production of the 16th century, clearly points to the so called Hunterian

---

* Oeuvres completes D'Ambroise Paré. Tom. 1, p. 372.

operation for aneurism; nor can the restitution of this honor to its legitimate owner detract from Mr. Hunter, whose fame rests too securely upon numerous, real, and valuable discoveries and improvements to need the support of that which belongs to others.

In the fall of 1814, Prof. D. "was applied to for assistance in a case of brachial aneurism, consequent to a wound received during the previous winter. * * * * Inasmuch as the parents of the youth were opposed to any surgical operation in the case, it was agreed to apply the common roller from the fingers to the shoulder, including thick compresses upon the aneurism, and upon the artery as high as the axilla." A cure was effected at the end of two months. "The satisfactory results from the treatment of this case, inclined me (Prof. D.) to the use of the same means in all aneurismal affections of the arteries upon the extremities, and for ten years in succession the results were equally successful and gratifying."

The manner of stating this case renders it apparent, that the Professor designs to make the impression that the treatment of aneurism, by compression, is original with him, and an accidental discovery. The Doctor is doubtless sincere and honest in this opinion, but a more familiar acquaintance with his illustrious predecessors would have taught him otherwise.

Guattani reports three cases of cure of popliteal aneurism by compression. His method consisted in covering the tumor with charpie, applying over this thick compresses, and one covering the track of the artery above the tumor. Over all he adjusted a bandage, making regular and moderate pressure; extending from the inferior part of the tumor to the superior portion of the limb.

In this method there was one serious inconvenience—the edematous engorgement of that portion of the limb below the bandage. To obviate this accident Genga, Freer, Scarpa, etc., proposed to apply the roller to the whole limb, beginning at the extremities of the fingers or toes, retaining the compresses upon the tumor and along the course of the diseased artery.

Here then is Professor D's treatment of aneurism in full, and these facts are alluded to by almost all modern writers on surgery; but yet they seem to have wholly escaped his notice.*

The Professor alludes to a number of cases treated by him after this method, but none of them are accompanied by a satisfactory detail of particulars, such as is calculated to fix the rules that are to govern in selecting the bandage in preference to the knife and ligature.

The influence of general medication over aneurisms is illustrated by cases and the inference deduced that constitutional treatment (diet, emetics, cathartics, etc.) acts in virtue of the control that is thus exerted over "local inflammation."

The Professor thinks he has rescued a "threatened victim" from aneurism of the left subclavian, by constitutional treatment; but the details of the case are so extremely meagre as utterly to preclude any judgment concerning the correctness of the diagnosis.

The following is a very extraordinary and interesting case, the successful management of which reflects honor on the operator.

"The patient, a laborious mechanic, had been an occasional and intense sufferer in his head for five or six years, for which much had been done in the way of local applications and internal remedies. Upon examination, the right eye was found to be remarkably protruded from the socket, with pulsations which could not fail to arrest the

---

* See Dict. de Med., vol. 2, p. 283. First Lines of the Practice of Surgery, by S. Cooper, vol. 1, p. 306. Dorsey's Surgery, 1813, vol. 2, p. 177. System of Surgery, by B. Bell, 1806, p. 109. Maladies Chirurgicales, par. M Boyer, tom. 2, p. 244. Nélaton, tom. 1, p. 457.

notice of the observing surgeon at some distance from the patient. The transverse suture at the outer corner of the eye was opened sufficiently to admit the end of the finger. A large portion of the os frontis, os temporis, and temporal plate of the sphenoid bone, and a part of the parietal bone were disjointed and elevated some lines above the proper level of an aneurismal affection of the internal carotid. The eye and ear of that side ceased to perform their functions." After the usual preparation the carotid was exposed and a ligature applied to it, "the patient immediately expressed himself as being relieved of a noise in his head which had been an attendant throughout the history of his malady."

"By the expiration of the second week from the operation, the tumor had receded so far that the disunited portions of the cranial bones had recovered their proper position. Before the conclusion of the month he left this place for St. Louis where he resumed, and is now engaged in prosecuting his occupation as a blacksmith."

The experience acquired in the constitutional management of aneurism led the Professor to the inference that the same method "would prove efficacious in the treatment of aneurismal affections of the heart." To establish the correctness of this conclusion several cases are given; but, unfortunately, they are not reported with such details as are necessary to satisfy the enquiring mind that there really existed an aneurismal affection of that organ; nor will this defect astonish any one when it is stated that Prof. Dudley does not recognise the value of auscultation and percussion in diagnosticating diseases of the heart.

The remainder of this, the first of Prof. Dudley's series, is occupied in the consideration of that favorite subject—the Bandage—a hobby which he has bestrode so long and eulogised so extravagantly, that one would think he was the first of mortals to discover its virtues, and, even to this day, the only one to appreciate its wonderful merits. In the present paper it is discussed as a great means of arresting hemorrhage; but we observe nothing valuable to note which may not be found in almost every surgical work from the earliest times to the present day. It would seem to be self-evident that, even prior to the date of the most ancient medical records, accident, and the instinct of self-preservation alone, must have taught the rudest savage the effect of pressure in arresting hemorrhage from divided vessels; yet whenever Prof. D. takes up this subject a tyro would imagine that compression was a novel remedy, and wonder at the imbecility of the old Masters who so unaccountably failed to discover and appreciate its magical properties. Indeed, not a few gentlemen have in times past gathered the honors of old Transylvania, and gone home in the full confidence that they had there learned the applicability of the bandage to the treatment of disease, which would ensure them a vast superiority over the graduates of any other institution. And yet, in every essential particular, it has been in as free use for ages as at the present day.

The Professor proceeds to inform his readers with due gravity, that "efficient compression, by means of bandage, so as to maintain immovably fixed the opposing sides of the vessels which have been divided in an operation, is a state of the parts in a wound incompatible with the loss of blood." This is doubtless a truism which no one can gainsay; for it must be evident that if the compression be sufficient to arrest the flow of blood the hemorrhage must cease.

Another principle, embraced in the following remarkable paragraph, does not appear to be quite so apparent—it may at least admit of question.

"Why ineffectual pressure will invite a hemorrhage, which subsides upon its removal, is not an appropriate subject for rational enquiry. As an elementary truth, it claims a place among others of like character from which flow those principles upon which all rational practice is founded."

This is the philosophy of the darkest ages of all science—not less repulsive than that which forbade Galileo to investigate the laws of motion that rule the planets; it is the spectacle of a philosophical teacher calling the attention of his pupils to a great practical phenomenon, but interdicting any inquiry into its cause. We may, at least be permitted to inquire why it "is not an appropriate subject for rational inquiry?" Whether it is invested with a character so holy as to render the inquiry sacrilegious? If after diligent inquiry, the "why" still eluded the search, would any harm result, and if discovered is it not extremely likely that some practical benefit would accrue to suffering humanity? The principle is ranked among "elementary truths;" but is no effort to be made to resolve it into more simple elements? Earth, air, fire and water were once elements with philosophers, and doubtless some of them would have denounced, as a visionary fanatic, any one who would have been rash enough to avow the purpose of seeking to analyse them. In every age of science there have been those who were strenuous in setting bounds to human thought and "rational inquiry." They are not wanting even at this enlightened day with all the wonders of modern discovery and invention staring them full in the face.

This school of philosophers have always proved hurtful to science, ever retarding its march and degrading its dignity. They content themselves with grasping the elements furnished by experience, forgetting that universal principle, constituting the very foundation of the Baconian philosophy, that every phenomenon has a cause. We know of no facts, either physiological or pathological, which "are not appropriate subjects for rational inquiry;" indeed, in any science no speculation, even, in regard to facts not perfectly comprehended, can be so absurd as not to be worthy of consideration. "The most remote and fanciful explanations of facts have often been found the true ones; and opinions which have in one century been the objects of ridicule, have in the next been admitted among the elements of our knowledge." Hence those who have penetrated farthest are least disposed to limit the researches of others. No one can behold the splendid achievements of alchemical and astrological researches without a feeling of repugnance to every effort to shackle the human mind in "rational inquiry."

Satisfy us, then, as to the fact that "inefficient pressure" *does invite* or increase hemorrhage, and we will not hesitate a moment to proceed to investigate its rationale; nor can we well conceive of a more appropriate subject for "rational inquiry." This dictum has been taught by Prof. Dudley for many years. *Is it true?* Does inefficient pressure invite hemorrhage? Or has the Professor only more fully established his claims to be ranked among the philosophers to whom we have alluded, and who are prone to award to error the devotion due only to truth, who begin by avowing a false principle, and end by restricting investigation?

We readily grant, as a truism, that if the compression be not sufficient to arrest the hemorrhage blood will continue to be lost. But does a certain degree of pressure, short of that which may be necessary altogether to arrest the hemorrhage, increase the quantity lost within a given time? We believe not. The principle, we feel convinced, has no foundation in fact. If Prof. Dudley should succeed in establishing its truth, he will have proved, that a reduction of the caliber of a tube will facilitate the passage of a fluid flowing from its orifice. Let any one place his finger upon a bleeding vessel and he will perceive that the hemorrhage therefrom is arrested in a direct ratio to the increase of pressure, and the consequent diminution of the caliber of the divided vessel. The truth is, ineffectual compression in cases of hemorrhage is objectionable, not because it increases an evil, but simply because it is ineffectual and consequently does not altogether abate it.

This first paper of the eminent Professor is extremely imperfect; it was evidently written without care as to matter, manner, or style, and is altogether unworthy of the fame of which he has been heretofore thought the deserving object. The paper abounds in generalities without system, and in practical deductions not warranted by the data given. The rigid and exacting character of our science demands something more than loose assertion unsupported by evidence. In our opinion, an author who pretends to publish a novel mode of treating disease is bound to furnish, in illustration of it, sufficient details to enable his readers to determine whether he was as correct in his diagnosis as he appears to be successful in his treatment. In this respect the paper before us is eminently defective. To give but a single example—the cases reported as "Diseases of the Heart," are not accompanied by the enumeration of a single symptom, by not the slightest proof that they were what they seemed to be; and yet every tyro in medicine can appreciate the necessity of such evidence in cases where sympathetic affections are so likely to be mistaken for organic lesions. It is possible they were of the nature of an aneurism; it is extremely probable that they were of a different character.

Respecting the style in which this paper is written, we will barely remark that it is often obscure, always inelegant, and generally incorrect. As to the latter defects they might be passed by, but we cannot perceive any good reason why the meaning of the author should not be expressed in intelligible language. We sincerely hope that the papers which are to follow may be written with more care, and approve themselves more worthy of commendation. C.

