# Errata

**Published:** 1849-12

**Authors:** 


### ERRATA.

Our collaborateur, who furnished us the review of Professor Dudley's paper on Aneurism, desires the correction of some typographical errors in his article. A portion of these errors, if errors they can be called, were made by us, in altering the quotation from Ambrose Paré to modern French. As soon as we saw that quotation in the words of Paré we felt twinges of the headache, for it called up reminiscences of a venerable old volume of Paré's Surgery, contained in the medical library of the Transylvania University, and which we had supposed no one had ever taken a weary journey through, except ourself. Between its barbarous orthography and obsolete French, the reading was a task of no ordinary magnitude, and we did not wish to inflict upon our readers even homœopathic doses of what had caused us so much suffering in allopathic doses. We accordingly dressed up old Ambrose in modern habiliments; but as our correspondent prefers to make him clatter in clogs, he shall be indulged.

The following errors are to be corrected:

On page 403, second line from bottom, for "their," read *this*.

The second word in the quotation from Paré should be *ie* instead of "le," and *ieune* should be substituted for "jeune- In the fourth line of the quotation, for "possera," read *passera*, and for "une," read *vne*.—Fifth line, for "un," read *vn*. In the seventh line, after the word "traitée," read *comme vne simple playe*. In the same line, for "say," read *soy*.

And on page 406, last line, read *Theden*, for Freer. This last error is not serious, for Freer made similar investigations to those made by Theden. B.

---

### DR. WELLS, THE AUTHOR OF THE "ESSAY ON DEW."

Prof. Bartlett recently delivered before the Medical Society in this city, a beautiful and eloquent lecture upon the character and merits of Dr. Wm. Charles Wells. The illustrious author of the "Essay on Dew," was an American, and is almost unknown to his countrymen. Kepler said Providence had to wait six thousand years for such a reader as himself, but Dr. Wells did not have to wait so long, and he found as good a reader of his merits, as Kepler was of "the starry dome."

We hope to present our readers with Dr. Bartlett's lecture in our next number.

